# Supplementary material for: How does social capital affect individual health among the elderly in rural China?—Mediating effect analysis of physical exercise and positive attitude
Source: PLoS One. 2020 Jul 27;15(7):e0231318. doi: 10.1371/journal.pone.0231318 (PMC7384663; doi:10.1371/journal.pone.0231318)
Supplement: Attachment — Submitted filename: Response to Reviewers.docx [file pone.0231318.s003.docx]

Response to Reviewers

Dear Dr./Prof. Frey,

On behalf of my co-authors, we thank you very much for giving us an opportunity to revise our manuscript, and we also appreciate the positive and constructive comments and suggestions, given by you and other reviewers, on our manuscript entitled “How does social capital affect individual health among the elderly in rural China?--mediating effect analysis of physical exercise and positive attitude” (ID:PONE-D-19-29520).

Having studied the reviewer’s comments carefully, we have considered your suggestions and comments, trying our best to make revisions which were marked in red in this paper. Please find the revised version enclosed, which we would like to submit for your kind consideration.

Point 1:Please address the minor issues raised by reviewer 3 regarding:the relationship between social capital and health outcomes.

**Response 1:**

We appreciate the comments of the reviewers. In our previous manuscript, we only discussed the relationship between structural and cognitive social capital during the literature review and data analysis process. In the discussion section of this revised manuscript, based on the results of empirical analysis, we conducted a more detailed discussion on the relationship between structural and cognitive social capital and health.

Firstly, we find that both structural social capital and cognitive social capital have a significant promotion effect on health, and the coefficients were 0.062 (p<0.01) and 0.097 (p<0.001), respectively. We explain the role of structural and cognitive social capital in promoting health (please see line 475-493).

Secondly, we find that the structural social capital's promotion of individual health among rural elderly is less than the cognitive social capital. We have explored this phenomenon in more depth. It may be because rural elderly rely more on mutual help and interpersonal trust in the community. They have more cognitive social capital than structural social capital, which has led to the different promotion of two types of social capital on individual health (please see line 496-509). We also find this difference exist in research from other regions. We analyzed our finding and results of other scholars, summarizing the reason. Rural elderly tend to develop cognitive social capital from community norm and trust, and they do not have a number of recreation facilities (e.g., exercise facilities, room for card games, etc) and organizations (e.g., elderly association, activity center for the elderly, etc) to get structural social capital (please see line 510-523).

Finally, we explore how two aspects of social capital connect with individual health (please see line 526-547). We found positive mentality and physical activity both play a mediating role on the relationship between social capital and health based on previous empirical research, but their mechanisms are different. This is probably because structural and cognitive social capital connect with health through different ways, and they affect different aspects of health. Structural social capital mainly promotes health through team members' access to health information and imitating health behaviors. Cognitive mainly relieve individual stress and regulate mindset to promote health (please see line 548-570)

We hope to discuss the above three parts to further analyze the relationship between structural social capital and individual health.

Point 2:Please address the minor issues raised by reviewer 3 regarding: greater detail about the population under study

**Response 2:**

Thanks a lot for the editor's suggestion. We apologize for not give detail introduction about the elderly in rural China. Older adults in rural areas constitute the majority of elderly persons in China, and a large number of them do not have much financial income, do not get adequate medical and health services, and often take care of themselves. The main problems rural elderly face are economic poverty, poor physical health, mental loneliness and etc. With the aging of rural areas, the health problem of rural elderly is more prominent than that of urban elderly, which need significant concern of the whole society (please see line 54-65).

The living environment of the elderly in rural China is special. The culture of rural China values trust, mutual assistance and reciprocal exchange, which provide cultural soil for cultivating social capital. Rural residents tend to be more altruistic, honest, and trusting of others, and they reported higher levels of civic cohesion and interpersonal trust than their urban counterparts in China. Under such circumstances, it is necessary and important to study social capital and individual health among the elderly.

We would like to express our great gratitude to you and other reviewers for the comments on our paper. Looking forward to hearing from you.

Thank you and best regards.

Yours sincerely,

Zhang Yue

Corresponding author:

Name: Zhang Yue

E-mail: [yuezhang.znufe@163.com](mailto:yuezhang.znufe@163.com)

Response to Reviewer 3 Comments

Dear Reviewers,

Thank you for your letter and for the reviewers’ comments concerning our manuscript entitled “*How does social capital affect individual health among the elderly in rural China?--mediating effect analysis of physical exercise and positive attitude*” (ID: PONE-D-19-29520). Those comments are all valuable and very helpful for revising and improving our paper, and simultaneously they are of great significance to give instructions to our research. We have studied the comments carefully and made revisions which we hope to meet with approval. Revised portions are marked in red in the original paper. The main revisions on this paper, along with our responses to the reviewer’s comments, are as follows:

**Point 1:**More specific in the section describing the evidence on the relationship between social capital in its structural and cognitive dimensions and different health outcomes.

**Response 1:**

We appreciate the comments of the reviewer. In previous manuscripts, we only introduced the relationship between social capital and health in the literature review and empirical research sections. We apologize for ignoring detailed explaining the relationship between social capital and individual health. We have improved the manuscript and focused on the discussion to further explain the relationship between social capital and individual health. Our analysis follows the following lines: Is there a relationship between structural social capital and individual health? What are the coefficients of structural and cognitive social capital on individual health? How do structural and cognitive social capital connect with individual health?

Firstly, we find that both structural social capital and cognitive social capital have a significant promotion effect on health, and the coefficients were 0.062 (p<0.01) and 0.097 (p<0.001), respectively. We explain the promotion of structural social capital. People who have structural social capital could get available public spaces and access to mutual support, and rapid diffusion of health information and healthy norms of behavior through their clubs and associations (please see line 475-487). We also explain the role of cognitive social capital in promoting health. The impact of cognitive social capital is mainly psychological support through interpersonal trust and mutual assistance, which generally predicts good self-rated health (please see line 485-493).

Secondly, we explained why the coefficient of cognitive social capital on individual health is larger than structural social capital. The elderly in rural China participated in far fewer social organizations than old adults in urban China. Trust and mutual assistance between neighbors are common in the social life of rural elderly, and their perception of neighborhood relations is stronger than that of organizational participation (please see line 496-509). We also find this difference exist in research from other regions and summarized the reason: rural elderly tend to develop cognitive social capital from community norm and trust, and they do not have a number of recreation facilities (e.g., exercise facilities, room for card games, etc) and organizations (e.g., elderly association, activity center for the elderly, etc) to get structural social capital (please see line 510-523).

Finally, we explored that how structural and cognitive social capital connect with individual health. We focused on two different ways in which social capital affects health. Structural social capital mainly provides the dissemination of health information and the imitation of health behaviors for the elderly, which demonstrated the mediating effect of physical exercise on the relationship between structural social capital and individual health was greater than positive attitude. Structural social capital is mainly beneficial to physical health, and cognitive social capital play a positive role in mental health. Cognitive mainly relieve individual stress and regulate mindset to promote health, which demonstrated the mediating effect of positive attitude on the relationship between cognitive social capital and individual health was larger than physical activity (please see line 548-570).

We hope to explain the different relationship between structural and cognitive social capital and individual health through discussion of these three aspects above.

**Point 2:**This part would benefit greatly if the authors could specify the population in which the studies had been conducted - epsecially taking into account that they argue that it has been little explored in elderly popultion from China.

**Response 2:**

We agree with the reviewer’s suggestion. In previous manuscripts, we did not give a detailed introduction to the group of elderly people in rural China. In fact, the health problems of this group are worthy of attention (please see line 53-65). We introduce this group as follows:

*Older adults in rural areas constitute the majority of elderly persons in China, and a large number of them live alone at home because their family numbers go out to work in cities with the acceleration of urbanization. These old people do not have much financial income, do not get adequate medical and health services, and often take care of themselves. The main problems rural elderly face are economic poverty, poor physical health, mental loneliness and etc. With the aging of rural areas, the health problem of rural elderly is more prominent than that of urban elderly, which need significant concern of the whole society. Currently, Chinese government has carried out “Healthy China” strategy to meet the challenge of aging. Under such circumstances, knowledge of social determinants of healthy aging are crucial for the development of evidence-based policies and interventions and the sustainable development of Chinese society.*

The environment in which they live is rich in social capital, and social capital is closely related to the lives of these elderly people. We explain the importance of studying the health and social capital issues of this group as follows :

*China is a typical Guanxi-based society, and evidence shows Guanxi and social capital have similar connotations and effects. Chinese tend to seeking for social support and maintain social status in the social structure in which they live. In addition, the culture of rural China values trust, mutual assistance and reciprocal exchange, which provide cultural soil for cultivating social capital. Rural residents tend to be more altruistic, honest, and trusting of others, and they reported higher levels of civic cohesion and interpersonal trust than their urban counterparts in China. Some relevant departments do not provide sufficient or formal credit systems for rural elderly, and the elderly often rely more heavily on the development of social capital in daily life.*

For more details, please see line 68-81.

We hope to explore the association between social capital and individual health among the elderly in rural area and make a reference for the implementation of the “Healthy China” strategy.

Thanks again for the reviewers' comments and suggestions!
